# The mechanism of short-term monocular deprivation is not simple: separate effects on parallel and cross-oriented dichoptic masking

**DOI:** 10.1038/s41598-018-24584-9

**Published:** 2018-04-18

**Authors:** Alex S. Baldwin, Robert F. Hess

**Affiliations:** McGill Vision Research, Department of Ophthalmology, McGill University, Montreal, Quebec Canada

## Abstract

Short-term deprivation of the input to one eye increases the strength of its influence on visual perception. This effect was first demonstrated using a binocular rivalry task. Incompatible stimuli are shown to the two eyes, and their competition for perceptual dominance is then measured. Further studies used a combination task, which measures the contribution of each eye to a fused percept. Both tasks show an effect of deprivation, but there have been inconsistencies between them. This suggests that the deprivation causes multiple effects. We used dichoptic masking to explore this possibility. We measured the contrast threshold for detecting a grating stimulus presented to the target eye. Thresholds were elevated when a parallel or cross-oriented grating mask was presented to the other eye. This masking effect was reduced by depriving the target eye for 150 minutes. We tested fourteen subjects with normal vision, and found individual differences in the magnitude of this reduction. Comparing the reduction found in each subject between the two masks (parallel vs. cross-oriented), we found no correlation. This indicates that there is not a single underlying effect of short-term monocular deprivation. Instead there are separate effects which can have different dependencies, and be probed by different tasks.

## Introduction

Recent studies have found that short-term monocular deprivation can shift ocular dominance to favour the deprived eye^1–6^. Typically, these studies deprive one eye by placing a patch over it for 120–150 minutes. The patching effect is elicited by either opaque (black) or translucent patches. The opaque patches deprive the eye of both luminance and visual form input. The translucent patches remove the form information from the input, with a small luminance reduction. The shift in ocular dominance is temporary. The peak effect is found immediately following patch removal. The subsequent decay varies between studies and methodologies. The effect is still measurable at 30 minutes after removal, and may last for several hours^7^.

An ocular dominance shift has also been shown both using dichoptic movie stimuli^8,9^ and using continuous flash suppression^10^. The dichoptic movie studies replaced the patching with a pair of video clips shown to the two eyes. These videos were processed independently to show different information to each eye. This was used to identify the cause of the deprivation effect. They demonstrated that the effect is driven by the loss of high spatial frequency information in one eye. Additionally, Zhou *et al*.^8^ were able to show that the effect is not orientation-specific. They tested a condition where horizontal information was preserved during the deprivation period. They then tested subjects on a task where stimuli would be processed by horizontally-tuned channels. Subjects still exhibited the patching effect even when horizontal input had not been deprived.

Previous studies have used two different categories of task to quantify the dominance shift. The original study by Lunghi *et al*.^1^ used a binocular rivalry task. In this task, incompatible stimuli are shown to the two eyes. The subject’s perception alternates between the two stimuli. The measure of dominance is calculated from the relative duration of time during which the subject reports seeing each stimulus. Other studies have used a phase combination task. This measures the contribution of each eye to a fused binocular percept^2,11^. Although the patching effect is found with both tasks, there have been discrepancies between the two types of study. An effect of exercise on the magnitude of the patching effect was found with binocular rivalry^12^ but not with the phase combination task^13^. It was originally reported that scrambling the phase of a dichoptic movie in one eye does not elicit the patching effect^8^. A later study however showed that this is true for the phase combination task, but that there is an effect with a binocular rivalry task^9^.

The ease of eliciting and measuring the patching effect makes it an attractive candidate to be an index of visual plasticity. This would be useful if the strength of the effect gave some general measure of the susceptibility of the visual system to plastic changes. The discrepancies found between results from different tasks suggest however that patching may cause multiple separable effects. In this case, we may consider different tasks to target different components. Although the rivalry and phase combination tasks have been the most frequent, other measures have been used to study the effect of patching^2,6,14^. We will return to these studies in the Discussion. For our purposes here, we used a dichoptic masking task to measure the patching effect. We measured contrast thresholds for detecting a stimulus shown to one eye. These can be elevated by the simultaneous presentation of a mask to the other eye^15^. Previous studies have explored the relationship between dichoptic masking and both binocular rivalry^16,17^ and binocular combination^18^.

The magnitude of dichoptic masking varies depending on the mask stimulus. The use of sinusoidal gratings for the target and mask allows these effects to be investigated. The strength of dichoptic masking depends on the spatial frequency^15,19,20^, orientation^20–22^, and phase^15,20^ of the mask. Generally, the more similar the mask is to the target, the greater the masking. These results have been explained by a model that combines separate effects with different tunings^20^. Results from neurophysiology also seem to align with a similar account^23–26^. Under both explanations, masking is made up of multiple processes. These are affected to different extents by the similarity between the target and mask.

In this study, we measure the strength of dichoptic masking from two different masks. We compare a parallel mask where the spatial properties of the stimulus are the same in the two eyes, against a cross-oriented mask where the mask stimulus is rotated to be orthogonal to the target. We then measure how each type of masking was affected by patching. When considering the binocular orientation-tuned simple cells in primary visual cortex^27^ we would expect the parallel mask to activate the same population of cells as the target. The cross-oriented mask, on the other hand, would activate a separate population of orientation-tuned cells from the target (though still able to interact with the detection of the target through suppression)^26^. These two masks enable us to explore different components of the patching effect using the same psychophysical procedure. This allows us to make a prediction. If short-term monocular deprivation affects only one aspect of interocular suppression then the measures from the two mask types should be correlated.

## Results

**Figure 1:**
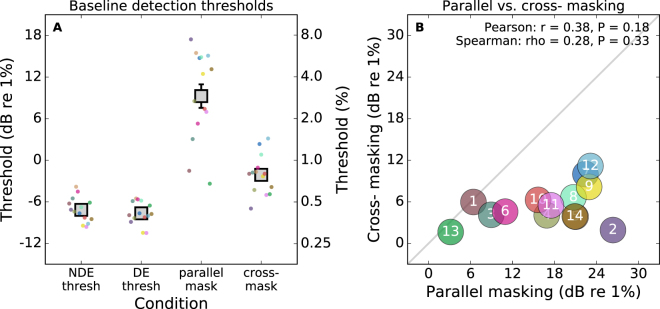
Panel A shows baseline detection thresholds averaged over 14 subjects. Coloured dots show data from individual subjects. Thresholds are given for the non-dominant (NDE) and dominant eyes (DE) without masking. Thresholds are also given for the dominant eye with parallel and cross-oriented 4% contrast masks in the other eye. Error bars show standard error of the mean. Panel B shows the relationship between the parallel and cross-oriented masking measured for each subject. The symbol for Subject 5 is obscured by that for Subject 14.

For the baseline measurements, contrast detection thresholds (without masking) were obtained. These were measured in both the dominant and non-dominant eyes. We determined which eye was which by using the Miles test for sighting dominance^28^, as described in the methods section. The contrast detection thresholds for the two eyes are presented in the left half of Fig. 1A. Thresholds without dichoptic masking were similar for the dominant and non-dominant eyes. The right half of Fig. 1A shows detection thresholds for stimuli presented to the dominant eye when a 4% contrast mask was presented to the non-dominant eye. With this dichoptic mask, thresholds in the dominant eye were elevated. In line with previous studies^20^, this elevation was more severe for the parallel mask (mean 16.9 dB, factor of 7.0) than for the cross-orientated mask (mean 5.6 dB, factor of 1.9). From the coloured dots showing the individual subject data (around each point in Fig. 1A) it is also apparent that there is more variability between subjects for the dichoptic masking conditions than for the thresholds without masking.

In Fig. 1B we explore the correlation between the parallel and cross-oriented dichoptic masking measured for each subject. For all subjects, the parallel mask had a stronger effect than the cross-oriented mask (though for the two subjects labelled “1” and “13” this difference was marginal). The range of masking effect magnitudes we find is surprising. For parallel masking, there is more than an eightfold difference in masking strength between the most affected and the least affected subjects. We expected that these individual differences may generalise between the parallel and cross-oriented mask conditions. There was however no significant relationship between the two types of masking (Pearson r = 0.38, P = 0.18; Spearman rho = 0.28, P = 0.33).

**Figure 2:**
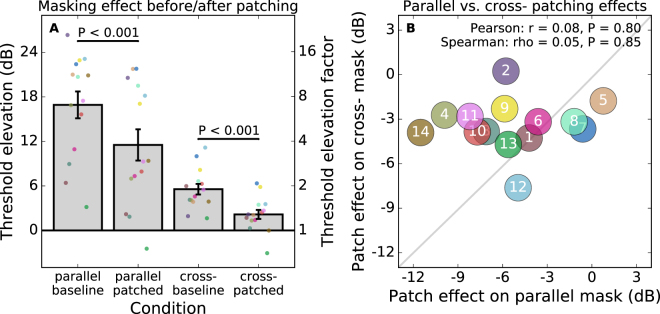
The effect of patching on the strength of dichoptic masking. Panel A shows the average threshold elevation from parallel and cross-oriented masks before and after patching. Coloured dots show data from individual subjects. In panel B the effects are plotted against each other for each subject, showing there to be no significant correlation.

We patched the dominant eye for each subject. The masking effects before and after 150 minutes of patching are presented in Fig. 2A. Masking is calculated as the threshold elevation that the mask introduces. This is relative to the threshold measured with no mask. We did not make additional measurements of unmasked detection thresholds after patching. Our pilot experiments had found no change from the baseline. For that reason, the unmasked thresholds from the baseline are used to calculate the masking for both the baseline and patched data. After patching, the threshold elevation from dichoptic masks with a parallel orientation decreased from 16.9 dB to 11.5 dB (from a factor of 7.0 to a factor of 3.8). For cross-oriented masks, threshold elevation decreased from 5.6 dB to 1.8 dB (from a factor of 1.9 to a factor of 1.2). To quantify the effect of monocular deprivation we took the dB difference between the masked thresholds before and after patching. The average patching effect for parallel masks was 5.4 dB, and for cross-oriented masks 3.8 dB. The patching effects were highly significant for both mask types (parallel: t(13) = 5.8, P < 0.001; cross-oriented: t(13) = 7.9, P < 0.001).

Regardless of whether the baseline levels of masking were correlated (Fig. 1B), we would expect the *change* in masking resulting from patching to be correlated if it resulted from changes in common underlying mechanisms (e.g. an effective decrease in the input gain for the eye that was not patched). We analysed the correlations between the parallel and cross-oriented mask effects for each subject (Fig. 2B). This tested whether the patching effect represented some general index of ocular dominance plasticity. Under that hypothesis, the individual differences would indicate some subjects to be “more plastic” than others. We would expect that those showing stronger effects of patching on one type of masking would also show stronger effects on the other. However, we in fact find no significant correlation between the patching effects measured with the two mask types (Pearson r = −0.13, P = 0.65; Spearman rho = −0.11, P = 0.71). It appears that the patching has separate effects on the strength of dichoptic masking from parallel and cross-oriented masks. This means that there is not a single underlying cause of the change in dichoptic masking we measure.

**Figure 3:**
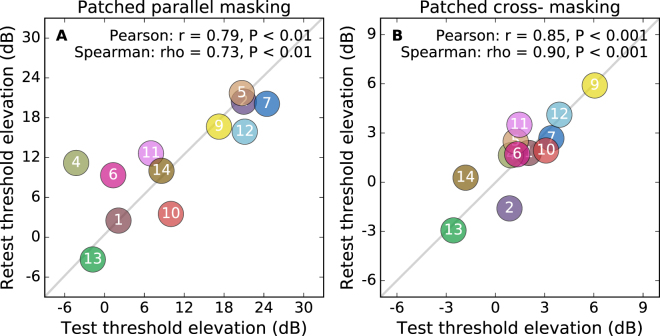
Test-retest reliability of the masked thresholds following patching. Panel A shows the correlation between the threshold elevation (relative to the baseline threshold) introduced by the parallel mask in the first and second testing sessions. Panel B shows the correlation between the measurements from the two sessions for the cross-oriented mask. Subjects 3 and 8 are omitted from this figure as they each repeated the experiment three times rather than twice.

We looked for explanations for the lack of a relationship between the effects on parallel and cross-oriented masking. One possibility would be if our measurements were highly variable. If subjects did not perform consistently on repetitions of the same task then that would affect our ability to show a correlation. One source of such a problem would be if the patching effect itself was not consistent between repeated sessions. To investigate this possibility, we analysed the test-retest reliability. We compared the data from the two testing sessions we conducted for each subject. This is shown in Fig. 3. The correlations between the first and second test sessions for the parallel dichoptic masking are shown in Fig. 3A. The two are significantly correlated (P < 0.01) with a good test-retest reliability (Pearson r = 0.79, Spearman rho = 0.73). The correlations for the cross-oriented masking are shown in Fig. 3B. This correlation is highly significant (P < 0.001) with very good test-retest reliability (Pearson r = 0.85, Spearman rho = 0.90). Additionally, the data from which thresholds are compared in Fig. 2B are combined over the two testing sessions that are being plotted separately in Fig. 3. For that reason, there will be a smaller measurement error associated with those values.

The slopes of the fitted psychometric functions were not normally-distributed (Shapiro-Wilk normality test, W = 0.95, p-value < 0.01). For that reason, we performed statistics on the log_2_ of the slope values (these were normal, W = 0.98, p-value = 0.14). A repeated-measures ANOVA performed across the six conditions was significant F(5,65) = 4.87, P < 0.001. We performed Holm-corrected t-tests for the pairwise comparisons. The only significant effects were between the patched parallel mask condition and the dominant eye threshold (t(13) = 4.00, P < 0.01), and the patched cross-oriented mask (t(13) = 3.22, P < 0.01) conditions. Shallower slopes in the parallel mask conditions are predicted by models featuring a nonlinear response to contrast^18,20,29^. This reduction occurs dichoptically because the mask contrast sums with the target contrast. This acts as a pedestal to turn the detection task into a discrimination task. Putting aside the conditions with the parallel masks, the mean log_2_ *β* across observers was 1.13 ± 0.08 (mean ± standard error). This converts to a slope of *β* = 2.2, a little shallower than previous findings^30–34^. With parallel masking, the slopes become more shallow. For the baseline measurement of the parallel mask condition (though not significant in the pairwise comparisons) the mean log_2_ *β* is reduced to 0.80 ± 0.50, converting to *β* = 1.7. For the parallel mask condition measured after patching, the log_2_ *β* was 0.21 ± 0.25. This gives a slope *β* = 1.2. Under the Two-Stage Model^18^ of binocular vision it is predicted that for different dichoptic mask levels the psychometric slope will first decrease and then increase again. The difference we find between the slopes for the baseline and patched conditions may therefore be due to a change in the effective strength of the mask.

## Discussion

We found clear effects of short-term monocular deprivation on dichoptic masking. The threshold elevation from both parallel and cross-oriented masks was reduced after patching. This reduction is consistent with the idea that the patching results in changes in cortical gain control that reduce the inhibition of inputs from the patched eye^4,5,10,35^. The magnitude of the effect of patching on the two types of masking varied between subjects. Within a subject however, the effects were of consistent magnitude between the two repetitions of the experiment. We did not find a correlation between the effects measured with the two mask types. Therefore, we conclude that patching has separate effects on the strength of parallel and cross-oriented masking.

Our results can be interpreted in the context of previous models of dichoptic masking. Baker & Meese^20^ proposed that the response to the target stimulus is suppressed by a dichoptic mask through two mechanisms. One mechanism is broadband across all orientations, and the other is tuned (Gaussian with σ = 67°). For parallel masks which are in the same phase, they report an additional masking effect arising from binocular summation. They refer to this as their *indirect effect*, which combines with the *direct effect* seen for masks in any phase. Therefore, our masking results with parallel gratings can be interpreted as including the combined effects of the broadband suppression, tuned suppression, and binocular summation. The results with the cross-oriented mask on the other hand will be dominated by the effects of broadband suppression. The broadband suppression effect would therefore contribute to masking for both mask types. This may explain the weak (non-significant) correlation reported in Fig. 1B. The correlation could result from individual differences in that broadband suppression. The correlation may be reduced by additional factors affecting the parallel mask. Large individual differences in the tuned suppression would explain the weak correlation.

We measured the patching effect by the reduction in masking seen after patching. We did not find a correlation between the patching effects found for parallel and cross-oriented masks. This demonstrates that the patching effect is complex. Within the Baker & Meese^20^ model, the broadband suppression can be affected separately from the two additional effects that account for the parallel suppression performance (the tuned suppression and summation components). Those two effects could be teased apart by a future study that manipulated the spatial phase of the mask. We can compare our methods to those used previously. The parallel condition would be most similar to the phase combination^2^ task. The cross-oriented condition, on the other hand, would be similar to the binocular rivalry task^1^. These relationships could be confirmed by a future study. We hypothesise that a correlation would be found in a study comparing parallel masking to the phase combination task (and also for cross-oriented masking and binocular rivalry). We also propose that the separation of these effects explains the differences found previously in studies that have used these two tasks^9,13^. It remains possible that other factors beyond the orientation of the gratings will affect the strength of the patching effect. For example, the spatial frequency used in this study (0.5 c/deg) is lower than that used in previous rivalry studies of the effect (e.g. 2–3 c/deg)^1,7,12^. We do not yet know whether the magnitude of the patching effect is correlated for the same task at different spatial scales.

Where other tasks have been used, it is possible to speculate as to which mechanisms of interocular suppression and combination they probe. The dichoptic surround suppression task^14^ should be related to parallel dichoptic masking. We expect this as surround suppression also shows spatiotemporal tuning^36–38^. However, surround suppression should not cause the additional masking effects that arise from binocular summation (reported by Baker & Meese)^20^. We therefore cannot say whether results from a parallel dichoptic masking task would correlate with those from dichoptic surround suppression. This returns us to the implications of our key finding. Our results indicate that the shifts in ocular dominance measured after short-term monocular deprivation are not due to a single simple effect. This aligns with previous evidence that manipulations of the paradigm can affect different tasks independently^9^. It is therefore possible that any plasticity effects observed may be task-specific. Future studies should be cautious about extrapolating from findings with a single task to assert a general effect on ocular dominance. Additionally, our result has implications for any possible therapeutic use of monocular deprivation (e.g. the treatment of amblyopia). Effects measured under specific experimental conditions may not generalise beyond them. Therefore, assessments of the clinical effectiveness of the deprivation should use a measurement that relates to a real-world improvement for the patient.

## Methods

### Equipment

Stimuli were presented on a gamma-corrected Clinton Monoray CRT monitor (800 by 600 pixels at 150 Hz). The mean luminance was 38 cd/m^2^. The viewing distance was 0.7 metres. At this distance, there were 27 pixels on the screen for each degree of visual angle. A ViSaGe setup (Cambridge Research Systems Ltd., Kent, UK) was used with FE-1 ferro-electric shutter goggles to achieve dichoptic stimulus presentation. The image on the monitor alternated between the stimuli for the left and right eyes on every refresh. This alternation was synchronised with the opening and closing of the shutters in the goggles to show the stimuli separately to each eye. Therefore, the refresh rate for each eye was 75 Hz. Previous measurements of the crosstalk of this equipment found that it had the lowest of any tested setup^39^. Showing a 100% contrast grating to one eye would result in only a 2% contrast crosstalk image in the other eye. The experiment and analysis software was programmed in Matlab 2010a (Mathworks, Natick, MA, USA).

### Stimuli

**Figure 4:**
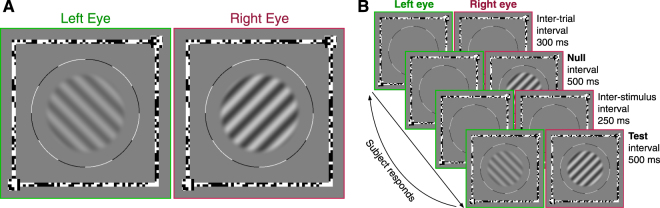
Panel A shows an example dichoptic pair of the stimuli used in this study. The two stimuli were presented separately to the two eyes using frame-interleaving. In this example, the target was a +45° oblique grating in the right eye. The dichoptic mask was a −45° oblique grating in the left eye. Panel B shows the sequence of stimuli shown in a single trial. In this example, the target was shown to the left eye in the second interval.

A pair of 0.5 c/deg sinusoidal gratings were used as stimuli (Fig. 4A). One grating was presented to each eye. The contrast of the gratings was modulated by a circular raised-cosine envelope. The width of the plateau was 5 degrees of visual angle. The contrast declined from unity at the edge of the plateau to zero over a distance of 1 degree. The phase of the gratings was the same in the two eyes. The temporal properties of the presentation were controlled by a Gabor function. The temporal frequency was 2 Hz and the duration sigma was 500 milliseconds. Therefore, in each presentation the gratings would visibly counterphase (black bars to white bars and white bars to black bars) once. We present stimulus contrast in log units (dB relative either to 1% contrast, or to thresholds from other conditions), calculated as

$${c}_{{\rm{dB}}}=20\times {\mathrm{log}}_{10}({c}_{ \% }).$$

A difference of 6 dB is roughly equivalent to a factor of two on the linear scale.

### Procedures

A two-interval forced-choice procedure was used to obtain thresholds for detecting a grating presented to one eye. Throughout the experiment a binocular fixation frame was presented to help the subject maintain their vergence. The stimulus location was indicated by a circle (Fig. 4). In these experiments, we obtained thresholds for detecting a monocular grating with no mask. We also measured thresholds for detecting a grating with a dichoptic mask. This mask was another grating presented to the other eye than the one receiving the target. This grating was fixed at 4% contrast, and either had the same orientation as the target (parallel) or a perpendicular orientation (cross-oriented). The target grating was oriented at either −45° for the left eye, or +45° for the right eye. The orientation of the target being detected was indicated at the beginning of each block with an example stimulus. Additionally, two squares were added to two of the corners of the fixation frame (bottom-left and top-right in Fig. 4A) to indicate to the subject the orientation of the target.

Stimuli were presented in two intervals (Fig. 4B). The inter-stimulus interval was 250 ms. In both intervals, the dichoptic mask grating was presented to the non-target eye. The target grating was presented only to the target eye in one interval. The subject then pressed one of two buttons to indicate whether the target was presented in the first or second interval. Subjects were then given auditory feedback as to whether their response was correct. The contrast of the target was controlled by a pair of staircases. One staircase had a 3-down-1-up rule, and the other a 2-down-1-up rule. They therefore converged at the 71% and 79% points of the psychometric function^40^. Although staircases were used for data collection, thresholds were obtained by fitting psychometric functions (see Analysis below).

**Figure 5:**
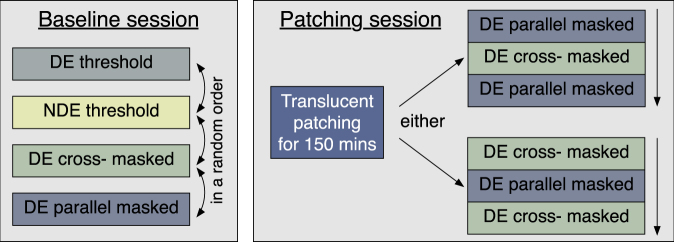
Sequence of conditions tested for the baseline and patching sessions. In the baseline sessions, the subjects were tested once on each condition in a random order. For the patching sessions, the subjects were tested on one of two fixed sequences of conditions.

For each subject, we chose the eye to be patched using the Miles test for sighting dominance^28^. The subject placed their hands together to form a peephole between the first finger and thumb of each hand. With both eyes open, they lined up this peephole at arms’ length with a target. They then alternately closed each eye. When the dominant eye was closed the target behind the peephole moved. The subject then reported which eye was dominant to the experimenter. The sequence of conditions tested is illustrated in Fig. 5. For the baseline measurements, subjects were tested on four different conditions in separate testing blocks. These were the detection thresholds for each eye, and the masked thresholds in the eye that would be patched with parallel and cross-oriented dichoptic masks in the unpatched eye. Each subject completed four baseline sessions (with either one or two sessions per day), therefore repeating each measurement four times.

To explore the effect of monocular deprivation, subjects completed two patching sessions. Subjects wore a translucent patch over one eye for 150 minutes. This patch deprived all form information from the outside world. The luminance received by the patched eye was also reduced slightly (by 20%). During the patching period, the subjects performed ordinary office tasks (e.g. reading or using a computer). After 150 minutes the patch was removed and the subject was immediately tested on three further testing blocks. These three blocks all measured masked thresholds. In different testing sessions, the sequence would either be parallel-cross-parallel or cross-parallel-cross (see Fig. 5). Most subjects (all except 3 and 8, see below) experienced both testing sequences (in a random order) over the two testing sessions. We test both sequence orders as the patching effect decays once the patch is removed. Counterbalancing in this way ensures that each mask condition is tested at each timepoint.

The exceptions to the order of testing outlined above were subjects 3 and 8, who were tested more extensively in the initial phase of the experiment. Overall each baseline for these subjects was measured either four (subject 3) or five (subject 8) times. These subjects were patched six times in total, with different combinations of conditions being tested in each patching session. The post-patch measurements for these subjects also included unmasked thresholds (where we saw no patching effect). Their post-patch measurements reported here were made three times in three separate patching sessions.

### Subjects

Fourteen subjects (including one of the authors) participated in the study (Table 1). Subjects wore their optical correction appropriate to the viewing distance for the experiment. All subjects gave written informed consent. The experiments were performed in accordance with the Declaration of Helsinki, and approved by the Research Ethics Board of McGill University Health Centre.

**Table 1:** Information of subjects participating in the experiment.

Subject#	Gender	Age	Dominant Eye	Left Eye Acuity	Right Eye Acuity
1	F	20	Right	20/20	20/20
2	M	33	Right	20/20*	20/20*
3	M	28	Right	20/20*	20/20*
4	F	22	Right	20/25	20/20
5	M	40	Right	20/20*	20/20*
6	F	20	Right	20/20*	20/20*
7	M	38	Left	20/20*	20/20*
8	M	30	Left	20/20	20/20
9	F	29	Left	20/20*	20/20*
10	M	23	Left	20/20*	20/20*
11	F	25	Right	20/20	20/34
12	F	25	Right	20/25	20/16
13	F	26	Right	20/20*	20/20*
14	F	25	Right	20/20*	20/20*

Acuities marked with an asterisk are for subjects who wore optical correction during the experiment.

### Analysis

Psychometric functions were fit by log-Quick functions in Palamedes^41^. For Figs 1 and 2 the data were collapsed over all repeats of the same condition from both testing sessions before fitting. For Fig. 3 the data from the two sessions were analysed separately. The lapse rate was fixed at 1%. Fitted psychometric function slope parameters were limited to a maximum value of *β* = 5. This changed the value from one of the eighty-four fits, where the original slope was very steep (*β* = 57). The correlation tests were performed in Python 2.7^42^ using SciPy^43^. The t-tests and ANOVA were performed in R^44^, using RStudio^45^.

### Data availability

Data available from figshare, 10.6084/m9.figshare.4879580.10.6084/m9.figshare.5742132.
